# Antimony(V) Adsorption and Partitioning by Humic Acid-Modified Ferrihydrite: Insights into Environmental Remediation and Transformation Processes

**DOI:** 10.3390/ma17174172

**Published:** 2024-08-23

**Authors:** Wei Ding, Shenxu Bao, Yimin Zhang, Bo Chen, Zhanhao Wang

**Affiliations:** 1 Key Laboratory of Green Utilization of Critical Non-Metallic Mineral Resources, Ministry of Education, Wuhan University of Technology, Wuhan 430070, China; dingwei@mails.swust.edu.cn (W.D.); zym126135@126.com (Y.Z.); hdwzhhao@163.com (Z.W.); 2 School of Resources and Environmental Engineering, Wuhan University of Technology, Wuhan 430070, China; 3 State Environmental Protection Key Laboratory of Mineral Metallurgical Resources Utilization and Pollution Control, Wuhan University of Science and Technology, Wuhan 430081, China

**Keywords:** antimony, iron (hydr)oxides, humic acid, complexes, adsorption capacity

## Abstract

Antimony (Sb) migration in soil and water systems is predominantly governed by its adsorption onto ferrihydrite (FH), a process strongly influenced by natural organic matter. This study investigates the adsorption behavior, stability, and mechanism of FH and FH–humic acid (FH-HA) complexes on Sb(V), along with the fate of adsorbed Sb(V) during FH aging. Batch adsorption experiments reveal that initial pH and concentration significantly influence Sb(V) sorption. Lower pH levels decrease adsorption, while higher concentrations enhance it. Sb(V) adsorption increases with prolonged contact time, with FH exhibiting a higher adsorption capacity than FH-HA complexes. Incorporating HA onto FH surfaces reduces reactive adsorption sites, decreasing Sb(V) adsorption. Adsorbed FH-HA complexes exhibit a higher specific surface area than co-precipitated FH-HA, demonstrating stronger Sb(V) adsorption capacity under various conditions. X-ray photoelectron spectroscopy (XPS) confirms that Sb(V) adsorption primarily occurs through ligand exchange, forming Fe-O-Sb complexes. HA inhibits the migration of Sb(V), thereby enhancing its retention within the FH and FH-HA complexes. During FH transformation, a portion of Sb(V) may replace Fe(III) within converted iron minerals. However, the combination of relatively high adsorption capacity and significantly lower desorption rates makes adsorbed FH-HA complexes promising candidates for sustained Sb adsorption over extended periods. These findings enhance our understanding of Sb(V) behavior and offer insights for effective remediation strategies in complex environmental systems.

## 1. Introduction

Antimony (Sb) finds extensive applications in plastic catalysts, flame retardants, and semiconductors, leading to significant quantities of Sb-containing substances being discharged into the environment [1,2]. Research indicates that approximately (4.7~47) × 10^6^ kg of Sb annually enters the soil through sewage [3,4]. While bearing resemblances to arsenic from Group 15, the geochemical behavior of Sb in natural settings is still inadequately elucidated [5,6]. Within natural environments, Sb can be found in multiple oxidation states (−3, 0, +3, and +5), with trivalent (Sb(III)) and pentavalent (Sb(V)) species being predominant in soils and sediments [7]. Sb is classified as a toxic and hazardous heavy metal element, with certain compounds known to induce toxicity and carcinogenic effects in humans [8]. Moreover, the US Environmental Protection Agency and the European Basel Convention have designated Sb as a priority pollutant [9,10]. Consequently, the issue of Sb contamination has garnered significant attention.

Sb mobility is frequently regulated by co-precipitation or adsorption interactions with iron-bearing minerals in numerous soil and sediment systems. Consequently, the dissolution and mineralogical transformations of host Fe(III) mineral phases, such as ferrihydrite (FH), goethite, and hematite, can profoundly impact the environmental fate and mobility of Sb [11]. These mineralogical alterations may lead to changes in the surface properties, such as surface area and surface charge, affecting Sb’s adsorption and desorption processes. Among the iron (hydrogen) oxides, FH is a widespread natural mineral. FH exhibits a strong affinity for binding with Sb(V) in aerobic soils and sediments, effectively restricting the migration and transformation of Sb(V) in soil and water environments [12,13].

FH is thermodynamically unstable and tends to transform into more stable phases, such as goethite and hematite. This transformation process is frequently observed in organic-rich wetland sediments and soils [14,15]. The rate and extent of this transformation, as well as the types of Fe(III)-bearing minerals formed, are influenced by several factors, including pH, the composition of the ligands, and the abundance of natural organic matter (NOM). These factors can affect the kinetics of mineral dissolution and precipitation reactions and the stability and morphology of the resulting Fe(III) minerals. The study by Namayandeh et al. demonstrated that oxygenated anions, such as nitrate (NO^3−^) and phosphate (PO_4_^3−^), influence the nucleation and growth of goethite and hematite during the transformation of FH [16]. Moreover, the presence of NOM can influence the speciation and complexation of iron and antimony species, potentially altering their adsorption behavior and mobility in soil and water environments [17]. Considering that the adsorption properties, such as affinity and specific surface area (SSA), of the resultant products following FH transformation (e.g., goethite and hematite) may vary from those of the original FH, the distribution of Sb between solid and dissolved phases could also impact the mineralogical transformation [18,19].

Humic acid (HA) is the major component of dissolved NOM in the natural aqueous system. NOM is a ubiquitous component present in most soils and natural water bodies. It demonstrates high chemical reactivity with iron oxides and can profoundly affect the transformation of iron minerals [20]. HA can closely bind with the hydroxyl groups of FH-forming complexes [21]. FH typically forms FH-HA complexes with HA through adsorption or precipitation. These FH-HA complexes serve as the fundamental structural units that influence the migration and transformation of pollutants in natural environments [22]. Compared to initial FH, FH-HA exhibits significant differences in pollutant adsorption capacity. Lu et al. observed that pure FH can adsorb 32% more Cu^2−^ from water compared to FH-HA [23]. Furthermore, FH co-precipitated with HA can absorb 21.6% more Pb(II) from water than pure FH, with Pb(II) forming a bidentate inner-sphere complex on the surfaces of FH and HA [24]. Sun et al. reported that the maximum adsorption capacities of Pb^2+^ and Cd^2+^ by the FH–synthetic humic-like acid composite were 87.41 mg/g and 47.10 mg/g, respectively [25]. These values represent 41.6% and 109.6% increases compared to those achieved by the FH–natural humic acid composite. The variance in adsorption capacity may stem from changes in the SSA of FH and the effective reaction sites of FH and HA resulting from their combination. Although the adsorption behavior of FH on Sb has been investigated, FH’s adsorption behavior and retention capacity after compounding with organic matter have rarely been reported. Therefore, exploring the adsorption behavior and retention capacity of FH-HA complexes for Sb is imperative, as it can yield valuable insights into the fate and conversion of Sb in natural systems. Given the widespread occurrence of FH-HA complexes in soils and sediments, elucidating their impact on Sb retention is essential for evaluating the risks associated with Sb contamination and devising efficient remediation approaches.

This study examines Sb(V) sorption on two distinct types of FH-HA complexes: those formed through co-precipitation with HA and those created by the adsorption of HA onto preformed FH surfaces. The adsorption kinetics and isotherm models were utilized to elucidate the dynamic sorption processes. Then, the interaction mechanism between Sb and FH-HA complexes was investigated through characterization analysis. Additionally, the stability of FH and FH-HA adsorption systems and the change in Sb(V) retention ability under the influence of environmental factors were studied. Further investigation was conducted to explore the impact of HA on the distribution of Sb and its redox speciation during FH conversion/recrystallization. The insights derived from this research contribute to a more comprehensive understanding of the interactions between FH, HA, and metals in soils and sediments, particularly in environments where HA interacts with iron (hydro) oxides and influences the migration of trace elements.

## 2. Materials and Methods

### 2.1. Synthesis of FH and FH-HA Complexes

Two-line ferrihydrite was prepared following the modified method of Karimian and Burton [18]. Humic acid (HA, FA ≥ 90% and carbon 422.7 g/kg) was purchased from Shanghai Aladdin Reagent Co., Ltd. (Shanghai, China). The carbon content of humic acid was measured by an Elementar Analysensysteme GmbH (Langenselbold, Germany) vario Macro cube. Potassium pyroantimonate (K_2_H_2_Sb_2_O_7_·4H_2_O, >99.0%, AR, Sinopharm, Beijing, China) was dissolved in ultrapure water (18.25 MΩ·cm) for the preparation of Sb in stock solution (1~100 mg/L). Iron trichloride hexahydrate ((FeCl_3_·6H_2_O, ≥99.0%, AR) was obtained from Sinopharm (Beijing, China).

For the co-precipitated FH-HA, 150 mL FeCl_3_∙6H_2_O (0.1 mol/L) was mixed with 20 mL of different concentrations of HA under vigorous stirring. The concentration HA solutions were 100 mg/L and 300 mg/L, respectively. The mixture was adjusted to pH = 7 and stirred continuously for 24 h. The obtained suspended solids were washed using dialysis bags to remove the residual ions. The samples were dried with a vacuum freeze-dryer and then finely ground and stored in the refrigerator for later use. The carbon content in the sample was determined by a vario EL cube elemental analyzer. The carbon content of the two co-precipitated FH-HAs was 19.20% and 30.53%. For the convenience of description, the two samples are abbreviated as FH-HA Cor-1 and FH-HA Cor-2, respectively.

For the adsorbed FH-HA, 1 g of FH powder was added to a solution containing a specific concentration of HA. The HA concentration was set as 100 mg/L and 500 mg/L, respectively. The pH of the FH-HA solution was adjusted to pH = 4.0 and incubated at 20 °C for 24 h. Finally, the FH-HA solution was centrifuged at 8000 r/min for 10 min to remove free HA. The obtained sample was freeze-dried. The carbon content of the two adsorbed FH-HAs was 0.9% and 1.6%, respectively. The two samples were abbreviated as FH-HA Adsr-1 and FH-HA Adsr-2, respectively.

### 2.2. Sb(V) Adsorption Experimental Designs

For adsorption edges spanning pH = 2–10, 0.01 g of FH or FH-HA complexes (0.4 g/L) was placed into Erlenmeyer flasks. Approximately 25 mL of a 50 mg/L Sb(V) solution was added to each flask and then placed on a shaker for 25 °C and 24 h. Following agitation, the suspensions were promptly passed through 0.45 μm syringe filters to retain the substrate adsorbed with Sb(V). Each adsorption experiment was conducted in triplicate for validation.

For adsorption kinetics (pH = 7 as a representative), precisely 0.01 g of FH or FH-HA complexes was introduced into a series of Erlenmeyer flasks, and a solution containing 25 mL of 50 mg/L Sb(V) was added. Samples were collected at regular intervals (0.25, 0.50, 1, 2, 3, 8, 24, 36, and 48 h).

For adsorption isotherms (pH = 7), FH or FH-HA complexes (0.01 g) were mixed with 25 mL of Sb(V) solution at concentrations of 1, 5, 10, 20, 50, 80, and 100 mg/L. The pH was adjusted and then the solution was reacted for 24 h.

### 2.3. Isothermal Desorption Experiments

In the analytical test, the substrate obtained through centrifugation in the isothermal adsorption experiment was added to 25 mL of ultrapure water, and desorption occurred at 150 r/min and 20 °C for 24 h. The same method was employed to determine the supernatant’s Sb(V) content, and the desorption rate was calculated.

### 2.4. Transformation Experiments

Transformation experiments were conducted using an abiotic batch incubation method based on Burton et al. [26]. Initially, 0.2 g of pre-adsorbed FH and FH-HA complexes was added to 50 mL of a buffer solution containing 0.05 M MES (2-(N-morpholino)ethanesulfonic acid), and adjusted to pH 7.0 with 0.1 M NaOH or 0.1 M HNO_3_. The samples were then incubated at 60 °C for the specified durations. The selected pH and temperature in this study were intended to expedite the transformation of FH to crystalline iron oxides [23]. The pH was monitored daily and adjusted to 7.0 ± 0.1 as needed. Conversion products were collected via centrifugation after 1, 5, 10, 20, 30, and 60 days, and the Sb(V) concentration in the filtered supernatant was measured.

### 2.5. Analytical Methods

Sb(V) concentration in diluted solutions was determined using inductively coupled plasma mass spectrometry (ICP-MS, PE Company 8300, New York, NY, USA). To investigate potential changes in the mineral structure of FH following HA loading, XRD analysis was performed using a D/MAX-III B instrument (Rigaku Co., Akishima, Japan). Measurements were taken within the 2θ range of 3–80°, employing Cu Kα radiation. Additionally, FTIR determined the samples’ functional groups and chemical bonds (Frontier, Denver, CO, USA). Surface area analysis of the samples was conducted using a pore size analyzer (Conta Autosorb-1MP, Giangarlo Scientific, Pittsburgh, PA, USA). The Zetasizer Nano ZS90 potentiometer (Malvern Panalytical Ltd., Malvern, UK) was used for zeta potential analysis. XPS analysis of the chemical states of elements in the measured samples was performed using an ESCALAB 250XI (Thermo Fisher Scientific, Waltham, MA, USA) (Al Kα X-ray at 1486.6 eV). All measurements were made at pressures below 10^−8^ Pa and used with a charge neutralization flood gun. In the XPS spectral data processing, all spectra were calibrated for binding energy using characteristic carbon (C 1s =284.8 eV). The Fe 2p, O 1s, and Sb 3d spectral curve was fitted using the XPS PEAK41 (V4.01).

## 3. Results and Discussion

### 3.1. Characteristics of FH and FH-HA Complexes

**Figure 1:**
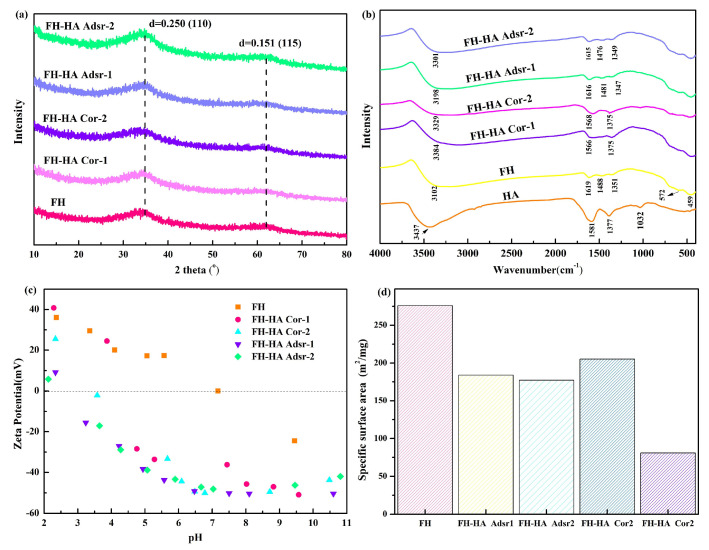
(**a**) XRD pattern, (**b**) FTIR spectra, (**c**) zeta potential, and (**d**) SSA analysis of FH and FH-HA complexes.

Figure 1a illustrates XRD patterns for the initial FH and FH-HA, showing two broad diffraction peaks at 35.89° and 61.34° and diffuse reflections that are typical of two-line ferrihydrite [27]. The absence of additional diffraction peaks indicates that there is no crystallization transformation when FH is combined with HA. This suggests that the interaction between FH and HA does not alter the amorphous nature of the FH, maintaining its non-crystalline structure.

The FTIR spectra presented in Figure 1b illustrate the broad spectral features of both FH and FH-HA, with prominent peaks observed at approximately 3200 cm^−1^ and 1619 cm^−1^, attributed to the stretching vibrations of hydroxyl groups, which constitute the fundamental functional group of FH [28]. A distinct red shift in the FH-HA spectrum is observed at 3200 cm^−1^ compared to FH. Moreover, an increase in peak intensity around 1619 cm^−1^ is noted with higher HA content. Previous studies have reported Fe-O and Fe-OH bending vibrations at 1488 cm^−1^ and 1351 cm^−1^, respectively [29]. Breaking Fe-O bonds imparts hydroxyl group coordination ability within the crystal structure. Additionally, FH-HA exhibits a combination of carbonyl groups and hydrogen bonding around 1615 cm^−1^, with the carbonyl group in carboxyl contributing to asymmetric stretching vibrations [30]. Distinctive absorption peaks of FH at 456 cm^−1^, 572 cm^−1^, and 1032 cm^−1^ are observed in both FH and FH-HA spectra, consistent with reports in the literature [31]. Upon FH-HA complexation, the peak intensity at 1488 cm^−1^ weakens, with a broader peak shape than FH. Of note, bands resembling those of HA emerged at 1581, 1377, 1032, and 3437 cm^−1^, with the 1581 cm^−1^ and 1377 cm^−1^ bands corresponding to the antisymmetric and symmetric vibration peaks of carboxyl groups [32]. Similarly, FH-HA complexes exhibit weakened carboxyl group symmetry vibration peaks and antisymmetric bands at 1377 cm^−1^ and 1581 cm^−1^ [33]. These findings suggest that -OH on the iron oxide surface undergoes ligand exchange reactions with the -COOH of HA, forming iron oxide–humic acid complexes.

The zeta potential of a solid surface is a critical property influencing adsorption behavior. As depicted in Figure 1c, the zeta potential data for FH and FH-HA gradually decrease with increasing pH. The point of zero charge (PZC) values for FH ranged from 6.0 to 8.0, whereas for the FH-HA complexes, the PZC values were notably shifted to the left. This shift may result from ionizing acidic functional groups in HA, which coat FH’s surface [34]. Interestingly, there appears to be no correlation between the change in zeta potential and the content of HA. This observation underscores the complex interplay between surface chemistry and pH-dependent interactions, indicating that factors beyond mere HA content contribute to the zeta potential variations.

The SSA of the adsorbent is a crucial indicator influencing adsorption efficiency. Figure 1d determined the SSA of both FH and FH-HA complexes. The SSA of pristine FH is measured at 275.97 m^2^/g. Upon adsorption of FH-HA complexes, the SSA decreased from 183.98 to 177.22 m^2^/g with increasing carbon content. In comparison, the co-precipitated FH-HA complex showed a more substantial reduction in SSA, with values dropping from 205.33 to 80.95 m^2^/g. This observation suggests that co-precipitated FH-HA complexes result in considerable coverage or blockage of the FH surface due to the filling of micropores by humic acid.

### 3.2. Adsorption Edges

**Figure 2:**
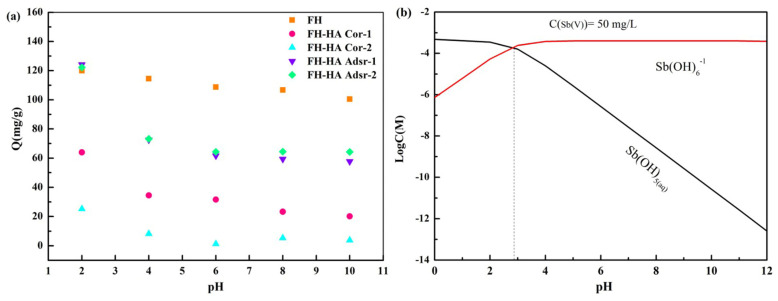
Effect of pH on Sb(V) adsorption by FH and FH-HA complexes (**a**) and logarithmic diagram of Sb(V) ion hydrolysis components (**b**).

Figure 2a illustrates that with increasing pH, the adsorption capacity of both FH and FH-HA complexes for Sb(V) decreases continuously. It is worth noting that pure FH shows a gradual decrease in adsorption across pH values ranging from 2 to 10. Conversely, FH-HA complexes exhibited a more rapid decline in Sb(V) adsorption at pH values below 6 than adsorbed FH-HA. Across a broad pH range, this observation suggests that the adsorption capacity of Sb(V) on both FH and FH-HA complexes may be primarily influenced by the surface charges of FH and FH-HA. As indicated by the analysis of Figure 2b, at pH levels around 3, Sb(V) predominantly exists in an anionic form.

Consequently, the positive charge on the complex’s surface leads to a strong electrostatic attraction between FH, FH-HA, and Sb(V) (Equation (1)).

Sb(OH)_5(aq)_ + H_2_O ↔ Sb(OH)_6_^−^_(aq)_ + H^+^ pK_a_ = 2.72 (1)

Figure 2b indicates that negatively charged species Sb(OH)_6_^−^ dominate the Sb(V) species within the pH range of 2.9 to 12. Hence, an increase in pH results in a greater repulsive force between the adsorbent and the Sb(V) anion, thereby reducing the amount of Sb(V) adsorption. Additionally, HA significantly diminishes Sb(V) absorption across the pH range of 2 to 12, with higher HA loading correlating with lower Sb(V) adsorption. However, the decrease in adsorption exhibits a nonlinear relationship with carbon content, suggesting that specific FH adsorption sites display a stronger affinity for Sb(V) than HA [35].

### 3.3. Adsorption Kinetics

**Figure 3:**
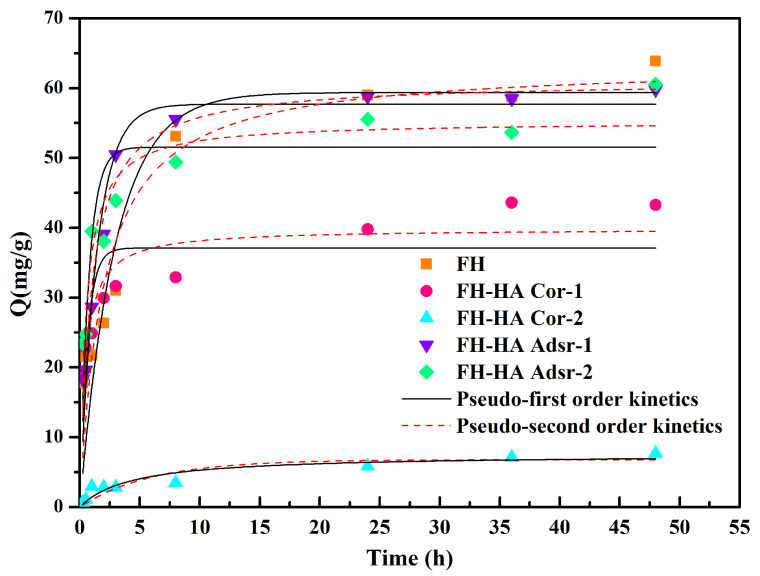
Time of Sb(V) adsorption onto FH and FH-HA complexes.

Figure 3 depicts the adsorption of Sb(V) on pure FH and FH-HA complexes, with equilibrium adsorption reached within 5 h. Both FH and FH-HA complexes possess abundant high-energy adsorption sites on their surfaces, facilitating rapid initial reaction with Sb(V) and subsequent increase in adsorption capacity. However, as the process progresses, adsorption capacity stabilizes, primarily relying on low-energy adsorption sites, resulting in a slower growth rate [36]. The two FH-HA complexes had a significant difference in the initial adsorption capacity. Co-precipitated FH-HA consistently exhibited lower adsorption capacity compared to adsorbed FH-HA. These findings suggest that available adsorption sites decrease when FH surfaces co-precipitate with HA.

To elucidate the fundamental mechanisms of the adsorption process, a pseudo-first-order kinetics (PFO) model (Equation (S2) in Supplementary Materials) and a pseudo-second-order kinetics (PSO) model (Equation (S3)) were employed to fit the adsorption equilibrium curve. The fitting results are presented in Table 1.

From Table 1, the correlation coefficients indicate a better fit for the PSO models than the PFO models. This suggests that the PSO kinetic model provides a more accurate representation of the adsorption process of Sb(V) on FH and FH-HA complexes. The adsorption of Sb(V) by FH and FH-HA complexes is predominantly governed by chemical adsorption, attributed to FH and HA’s relatively high surface activity, along with their adsorption performance and coordination ability [37]. Notably, neither the adsorption nor co-precipitation of FH and HA altered the binding mechanism of FH with Sb(V). It has been reported that when the adsorbent reacts with the adsorbate to form a chemical or covalent bond, the functional groups of both the adsorbate and the adsorbent typically form inner surface complexes [38]. From this, it can be inferred that FH and FH-HA complexes react with Sb(V) to form inner surface complexes. In addition, the equilibrium adsorption amounts calculated from PSO kinetics demonstrate that higher concentrations of HA result in a weaker adsorption capacity of FH for Sb(V).

### 3.4. Adsorption Isotherms

**Figure 4:**
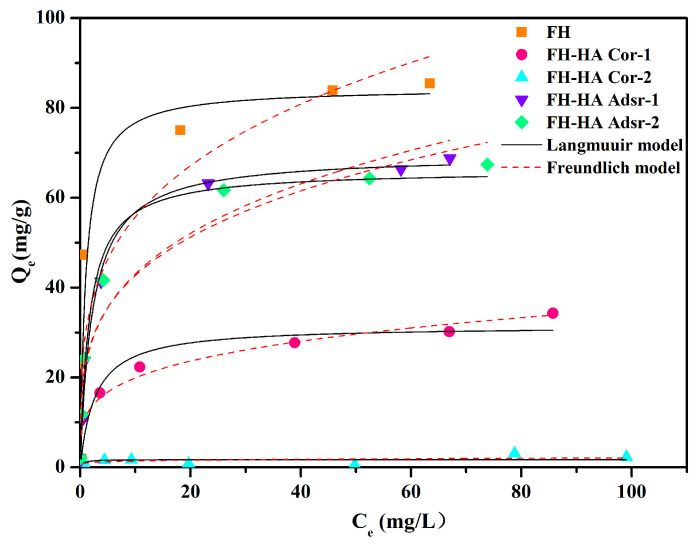
Adsorption isotherms of Sb(V) on FH and FH-HA complexes.

Figure 4 illustrates a decrease in the adsorption capacity of Sb(V) by FH-HA complexes compared to FH. The adsorption strength of adsorbed FH-HA for Sb(V) is greater than that of co-precipitated FH-HA. Additionally, the equilibrium adsorption amount of Sb(V) shows slight variation with increasing HA loading. The observed reduction in adsorption capacity and binding affinity implies a significant inhibition of Sb(V) adsorption onto FH surfaces by HA. Moreover, this inhibition effect intensifies with increasing organic carbon (OC) loading.

To comprehensively analyze Sb(V) adsorption on FH and FH-HA complexes, the equilibrium adsorption data were fitted to the Freundlich (Equation (S4)) and Langmuir (Equation (S5)) isotherm models. The high goodness of fit observed in Table 2 indicates the suitability of the Langmuir model for simulating Sb(V) adsorption onto FH and FH-HA complexes, suggesting a monolayer adsorption mechanism. A distinct variation in adsorption behavior exists between FH and FH-HA complexes. Specifically, co-precipitated FH-HA demonstrates a lower affinity for Sb(V) than adsorbed FH-HA and FH. The adsorption capacity of the complexes decreases as the concentration of HA increases. Variations in the adsorption behavior may be due to differences in the SSA of the minerals. Previous studies have highlighted a positive correlation between SSA and maximum adsorption capacity (*Q_max_*) [39].

### 3.5. Desorption Isotherms

**Figure 5:**
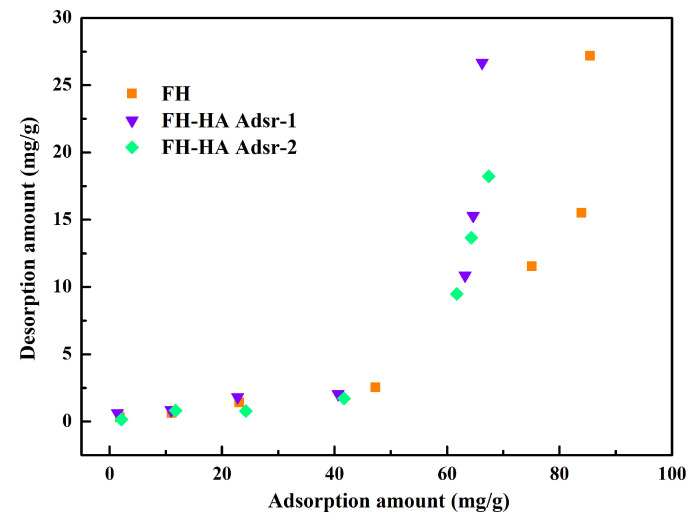
FH and adsorbed FH-HA desorption Sb(V) isotherms.

In Figure 5, within the adsorption range of 0 to 50 mg/g, both FH and adsorbed FH-HA exhibit low desorption capacities, while desorption testing for co-precipitated FH-HA was not conducted due to its low adsorption capacity. As the adsorption amount increases, it can be observed that adsorbed FH-HA exhibits a low-level desorption rate of Sb(V) compared to FH. This observation suggests that adsorbed FH-HA possesses strong retention capabilities for Sb(V). The lower desorption rate at lower concentrations is attributed to the predominant obligate adsorption of Sb(V), primarily involving the high adsorption sites on the surfaces of FH and adsorbed FH-HA [40]. Additionally, the effects of HA and metal ions include surface adsorption and complexation, with metal ions adsorbed by HA exceeding the amount present in HA itself. As equilibrium adsorption concentrations increase, the retention capacity of FH and adsorbed FH-HA for Sb decreases, leading to an increased release capacity. It is worth noting that even as the adsorption amount increases, the desorption rate remains below 35%, indicating relatively stable Sb(V) adsorption by adsorbed FH-HA. Upon reaching adsorption–desorption equilibrium, the majority of Sb remains within the adsorbent. This stability can be ascribed to the high affinity of FH surfaces with Sb(V), forming internal phase bidentate binuclear coordination compounds through coordination bond exchange reactions, which renders desorption challenging [40].

### 3.6. Effect of Transformation on Antimony Adsorption

FH demonstrates poor thermodynamic stability, often transforming into iron oxide with increased crystallinity through recrystallization. HA exhibits a strong affinity for mineral surfaces and plays a crucial role in mineral transformations [41]. Therefore, exploring the stability of the FH and adsorbed FH-HA adsorption system and the changes in Sb(V) retention ability under diverse environmental conditions is critical.

**Figure 6:**
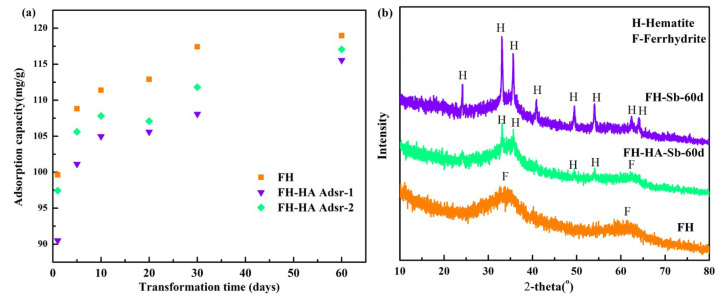
Transformation adsorption behavior of FH and adsorbed FH-HA ((**a**), 60 °C, pH = 7, initial C(_Sb(V)_) = 50 mg/L) and XRD patterns of the transformation products (**b**).

In Figure 6a, it is observed that during the initial phase of transformation (0–10 days), the retention capacity of both FH and adsorbed FH-HA for Sb(V) markedly increases with prolonged transformation time. As the conversion progresses, the retention trend of Sb(V) by FH and adsorbed FH-HA gradually stabilizes. This suggests a process where FH undergoes conversion, with Sb(V) partially adsorbed onto the iron oxide surface and replaced by entry into the crystal lattice or Fe structure [19]. Consequently, the formed iron oxide becomes structurally fixed, increasing Sb(V) adsorption capacity under conditions of high Sb(V) concentration. As shown in Figure 6b, under conditions of pH = 7, 60 °C for 60 days, both with and without the presence of HA, FH underwent conversion to hematite. Interestingly, HA was observed to partially inhibit the phase transition rate of FH to hematite during transformation, resulting in a relatively higher content of FH in the transformation products. This inhibition effect could be attributed to the complexation of HA with FH, leading to its adsorption onto the FH surface [42]. This process inhibits FH hydrolysis and prevents the formation of crystal nuclei, thereby impeding the transformation into iron oxides such as hematite.

### 3.7. *Adsorption Mechanism*

**Figure 7:**
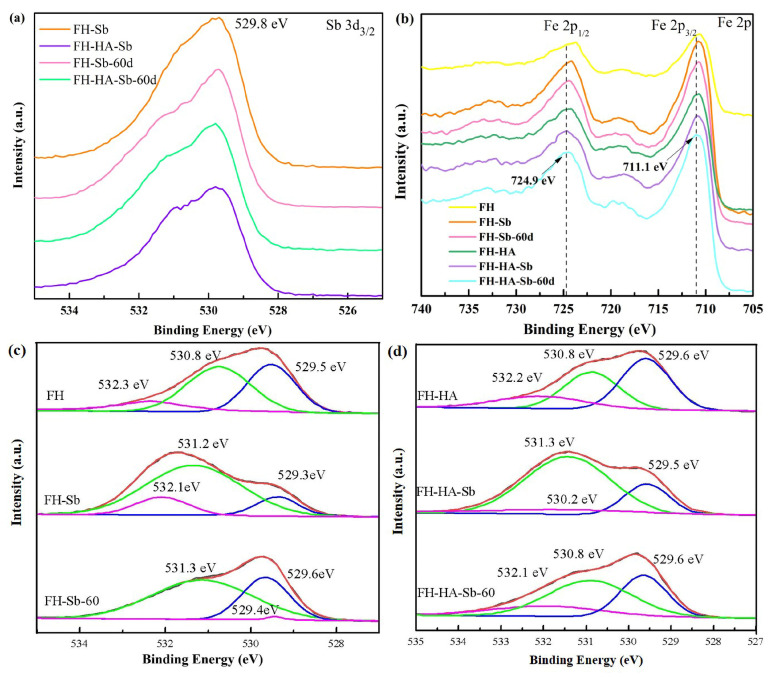
XPS spectra of FH and adsorbed FH-HA: (**a**) Sb 3d, (**b**) Fe 2p, and (**c**,**d**) O 1s.

Figure 7a presents the Sb 3d XPS spectra of both FH and adsorbed FH-HA. The high-resolution XPS signal corresponding to a binding energy of 529.8 eV was attributed to Sb 3d_3/2_, further confirming the Sb(V) adsorption on the FH surface [43]. Additionally, Figure 7b depicts the Fe 2p XPS spectra; the corresponding binding energies are 723.6 eV and 710.7eV, respectively, showing that Fe is +3 valence [44]. Upon adsorption of Sb(V), the peak positions of Fe 2p_1/2_ and Fe 2p_3/2_ shifted to higher energy states (FH-Sb: 724.2 eV and 710.8 eV, FH-HA-Sb: 724.7 eV and 711.0 eV) for both FH and adsorbed FH-HA, indicating a strengthened interaction between Sb(V) and FH [45]. Remarkably, in the transformation products of FH-Sb-60d and FH-HA-Sb-60d, the Fe 2p_1/2_ and Fe 2p_3/2_ peak positions shifted to even higher energy states (FH-Sb-60d: 724.3 eV and 710.9 eV, FH-HA-Sb-60d: 724.9 eV and 711.1 eV) compared to the adsorption products. This shift suggests a further enhancement of the interaction between FH and Sb(V) after transformation, correlating with the observed increase in the amount of Sb(V) adsorbed post-transformation.

From Figure 7c,d, the peaks observed at 529.5, 530.8, and 532.3 eV in the O1s spectrum represent metal oxide lattice oxygen bonds (Fe-O-Fe), hydroxyl bonds (Fe–O-H or Fe-O-Sb), and oxygen in water molecules (H-O-H), respectively [46,47]. From Table S1, it is observed that after Sb(V) adsorption, the component at 531.2 eV increases from 44% to 78% for FH-Sb and from 47% to 74% for FH-HA-Sb, suggesting that a portion of -OH groups interact with Sb(V), possibly through ligand exchange (-O-Sb), thereby intensifying the peak intensity with this new species. Hence, surface ferric hydroxyl groups are pivotal agents in antimony formation on ferrihydrite surfaces, consistent with prior spectral studies [48]. Unfortunately, the close binding energies of -O-H and -O-Sb in FH-Sb and FH-HA-Sb hinder the differentiation of their relative contributions [49]. In the conversion products FH-Sb-60d and FH-HA-Sb-60d, the peak at binding energy 532.3 eV (Fe-O-H) is either absent or very weak, attributed to the dehydroxylation of FH into hematite during the conversion process. The comparison shows that HA can inhibit the conversion of FH into hematite. The peak areas corresponding to binding energies of 531.3 eV and 529.6 eV, respectively, increase significantly, suggesting that Sb(V) can be embedded into the secondary iron mineral structure by substituting Fe(III) in the form of co-precipitation, forming an Fe-O-Sb structure and Fe(III)-Sb(V) co-precipitation [50,51].

**Figure 8:**
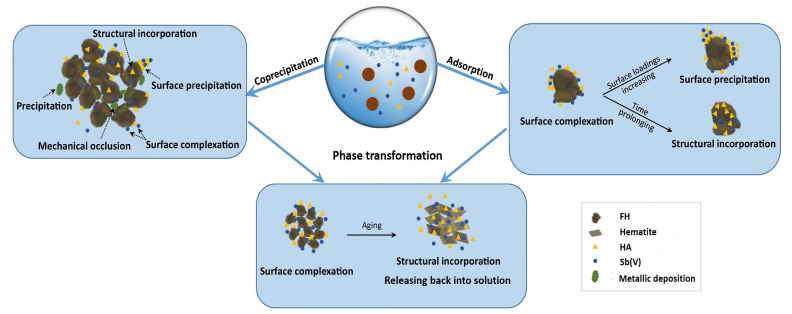
Adsorption mechanism of Sb(V) during adsorption and transformation of FH and FH-HA complexes.

In summary, the primary adsorption mechanism can be categorized into two components, as depicted in Figure 8. The adsorption of Sb(V) by FH and FH-HA complexes primarily occurs through ligand exchange, resulting in the formation of Fe-O-Sb inner/outer spherical complexes in edge-sharing and biangular-sharing modes [52]. Additionally, electrostatic attraction may also contribute to the adsorption of Sb(V) on FH and FH-HA complexes. During the FH phase transformation process, Sb(V) in solution continuously reacts and combines with FH and its transformation products. Under favorable conditions, a notable quantity of Sb(V) will adhere to the surface of iron minerals through electrostatic attraction. Some will be bound to the surfaces of hematite transformation products through surface co-precipitation (FeSbO_4_), while others will form inner spherical double-toothed double-nuclear coordination with these iron minerals through surface complexation mechanisms. These mechanisms may involve hydrogen bonding or outer spherical single-toothed complexation [53,54]. Furthermore, a portion of Sb(V) may replace Fe(III) within crystal structures of converted iron minerals such as goethite and hematite during FH transformation processes [55]. However, HA loading onto the surfaces of FH and hematite transformation products via chemical bonding reduces the SSA of iron oxide, which occupies active sites on their surfaces, leading to electrostatic repulsion towards Sb(V), thereby significantly inhibiting its adsorption by FH and transformation products [56,57,58].

## 4. Conclusions

This study examined the adsorption behavior, stability, and mechanism of FH and FH-HA complexes on Sb(V), as well as the fate of adsorbed Sb(V) during FH aging. The findings indicate that combining FH with HA does not alter its crystalline structure. Initial pH and concentration significantly influenced Sb(V) sorption, with lower pH levels decreasing adsorption and higher concentrations enhancing it. Sb(V) adsorption increased with prolonged contact time, with FH showing a higher adsorption capacity than FH-HA complexes. The incorporation of HA reduced reactive adsorption sites, increased negative surface charge, and decreased Sb(V) adsorption. Adsorbed FH-HA complexes exhibited higher SSA and stronger Sb(V) adsorption capacity across a wider pH range and different Sb(V) concentrations than co-precipitated FH-HA. XPS data confirmed that Sb(V) adsorption primarily occurs through ligand exchange, forming Fe-O-Sb complexes in edge-sharing and biangular-sharing modes. Additionally, electrostatic attraction also facilitates the adsorption of Sb(V).

The results of the transformation research show that HA inhibits the migration of Sb(V), contributing to its retention within the FH and FH-HA complexes even at equilibrium. During FH transformation processes, a portion of Sb(V) may replace Fe(III) within the crystal structures of converted iron minerals such as goethite and hematite. HA loading onto the surfaces of FH and transformation products reduced the SSA of iron oxide, occupying active sites and leading to electrostatic repulsion, thereby significantly inhibiting Sb(V) adsorption. However, the combination of relatively high adsorption capacity and significantly lower desorption rates makes adsorbed FH-HA complexes promising candidates for sustained Sb adsorption over extended periods. These findings enhance our understanding of Sb(V) behavior in complex environmental systems and provide valuable insights for developing effective remediation strategies.

## Figures and Tables

**Table 1:** Adsorption kinetic parameters of Sb(V) by FH and FH-HA complexes.

Adsorbent	PFO Kinetics			PSO Kinetics		
	*q*_e_ (mg/g)	*K*_1_	*R* ^2^	*q*_e_ (mg/g)	*K*_2_	*R* ^2^
FH	59.36	0.34	0.853	63.45	0.0079	0.900
FH-HA Cor-1	37.09	1.62	0.640	39.84	0.056	0.843
FH-HA Cor-2	6.77	0.16	0.824	7.53	0.031	0.884
FH-HA Adsr-1	57.70	0.71	0.950	61.03	0.018	0.973
FH-HA Adsr-2	51.55	1.34	0.760	55.20	0.035	0.903

**Table 2:** Adsorption isotherm parameters of Sb(V) by FH and FH-HA complexes.

Sample	Langmuir			Freundlich		
	*Q* (mg/g)	*K* (L/mg)	*R* ^2^	*K_f_* (mg^1−*n*^·L*^n^*/g)	*n*	*R* ^2^
FH	84.45	0.99	0.903	30.07	3.73	0.892
FH-HA Cor-1	31.47	0.37	0.948	11.27	4.05	0.896
FH-HA Cor-2	1.73	1.95	0.862	0.944	5.84	0.755
FH-HA Adsr-1	69.55	0.44	0.989	23.26	3.79	0.932
FH-HA Adsr-2	66.17	0.60	0.984	22.79	3.62	0.926

## Data Availability

The original contributions presented in the study are included in the article and Supplementary Materials, further inquiries can be directed to the corresponding authors.

## Supplementary Materials

The following supporting information can be downloaded at https://www.mdpi.com/article/10.3390/ma17174172/s1, Table S1: the relative proportion of O 1s for FH, FH-HA complexes and conversion products; Text S1: calculation equation; Text S2: calculation equation; Text S3: calculation equation.
