# Supplementary material for: Antimony(V) Adsorption and Partitioning by Humic Acid-Modified Ferrihydrite: Insights into Environmental Remediation and Transformation Processes
Source: Materials (Basel). 2024 Aug 23;17(17):4172. doi: 10.3390/ma17174172 (PMC11396405; doi:10.3390/ma17174172)
Supplement: Supplementary file 1 [file materials-17-04172-s001.zip › materials-3137088-supplementary.pdf]

Antimony(V) Adsorption and Partitioning by Humic Acid Modified Ferrihydrite: Insights into Environmental Remediation and Transformation Processes

Wei Ding[1, 2] Shenxu Bao[1, 2,*] Yiming Zhang[1, 2, 3] Bo Chen[1,2,*] Zhanhao Wang[1,2]

[1] Key Laboratory of Green Utilization of Critical Non-Metallic Mineral Resources, Ministry of Education, Wuhan University of Technology, Wuhan 430070, China

[2] School of Resources and Environmental Engineering, Wuhan University of Technology, Wuhan 430070, China

[3] State Environmental Protection Key Laboratory of Mineral Metallurgical Resources Utilization and Pollution Control, Wuhan University of Science and Technology, Wuhan 430081, China

* Correspondence: sxbao@whut.edu.cn (S.B.); bochen2012@whut.edu.cn (B.C.)

TableS1. Calculation equation.

The adsorption of Sb(V) was quantified by comparing the initial solution concentration with that of the equilibrium solution. The calculation was performed using the formula depicted in Eq. (1).

$$q_e = \frac{(C_0 - C_e)V}{M} \tag{S1}$$

where q_e ($mg \cdot g^{-1}$) is the amount of adsorption in equilibrium; V (mL) is the volume of the suspension; C_0 ($mg \cdot L^{-1}$) and C_e ($mg \cdot L^{-1}$) are the initial and adsorption equilibrium concentrations of Sb(V) in solution, respectively; M (g) is the mass of the adsorbent.

TableS2. Calculation equation.

$$q_t = q_e(1 - e^{-k_1 t}) \qquad \text{(S2)}$$

$$q_t = \frac{k_2 q_e{}^2 t}{1 + k_2 q_e t} \qquad \text{(S3)}$$

where q_e (mg·g^{-1}) is the adsorption amount of Sb(V) by an adsorbent at equilibrium; q_t (mg·g^{-1}) is the adsorption of Sb(V) at time *t*; k_1 and k_2 are equilibrium constants of adsorption rate.

TableS3. Calculation equation.

$$q_e = \frac{Q_{\max} \times kC_e}{1 + kC_e} \tag{S4}$$

$$q_e = k_f \times C_e^{\frac{1}{n}} \tag{S5}$$

where q_e is the adsorption amount of Sb(V) in the adsorbent, ($mg \cdot g^{-1}$); Q_{max} is the saturated adsorption amount of Sb(V) in the adsorbent, ($mg \cdot g^{-1}$); K and K_f are the equilibrium constants of Langmuir and Freundlich; C_e ($mg \cdot L^{-1}$) is the concentration of Sb(V) in the equilibrium solution and n is the coefficient of experience.

Table S1. The relative proportion of O 1s for FH, FH-HA complexes and conversion products.

**Samples**	**Fractions**		
	Oxygen bonds (Fe-O-Fe)	Hydroxyl bonds (Fe–O-H or Fe-O-Sb)	Oxygen in water molecules (H-O-H)
FH	40.3	44	15.7
FH-Sb	9.8	78	12.2
FH-Sb-60	33.6	65	1.4
FH-HA	50.2	47	2.8
FH-HA-Sb	25.5	74	0.5
FH-HA-Sb-60	39	58.7	2.3
